# Effect of impairment on health-related quality of life in people with multiple sclerosis: association of functional systems and EQ-5D-5L index values in a cross-sectional study

**DOI:** 10.1007/s11136-025-03928-9

**Published:** 2025-03-06

**Authors:** Richard Schmidt, Andreas Starke, Natalie Bednarz, Florian Then Bergh

**Affiliations:** Department of Neurology, Medical Faculty, Leipzig University, Liebigstr. 20, 04103 Leipzig, Germany

**Keywords:** Quality of life, Multiple sclerosis, Impairment, Disability, Cognition

## Abstract

**Introduction:**

Multiple sclerosis (MS) results in physical and cognitive impairments that negatively affect health-related quality of life (HRQoL). It is unknown to what extent the impact of MS-related impairments on HRQoL are reflected in the association of Expanded Disability Status Scale (EDSS) Functional Systems (FS) scores and EQ-5D-5L index values.

**Methods:**

This cross-sectional, single-center cohort study recruited people with MS (pwMS) attending an outpatient clinic at a German university hospital. Impairment was assessed via FS scores during routine visits. HRQoL was measured with EQ-5D-5L index values. The association of each FS score with EQ-5D-5L index values and the additive effect of all FS on EQ-5D-5L index values was modeled with multivariate linear regression (MLR).

**Results:**

Analyzing 115 participants, unadjusted MLR of single FS revealed that brainstem, pyramidal, cerebellar, sensory, and cerebral/cognitive dysfunctions were significantly associated with lower HRQoL. In MLR of all FS adjusted for covariates, a one standard deviation decrease in cognitive function was significantly associated with a 6% reduction in HRQoL.

**Conclusion:**

Dysfunctions in FS contribute to a decrease in HRQoL. Cognitive dysfunction was identified to maintain negative association with HRQoL after adjustment for covariates, and routinely assessed FS scores appeared useful indicators to identify pwMS who may benefit from comprehensive cognitive evaluations. This study adds to the growing body of evidence emphasizing the crucial role of cognitive function in HRQoL of pwMS and highlights the need for effective screening and therapeutic strategies.

**Supplementary Information:**

The online version contains supplementary material available at 10.1007/s11136-025-03928-9.

## Introduction

Multiple sclerosis (MS) is a chronic autoimmune, inflammatory and neurodegenerative disease that affected approximately 2.8 million people globally in 2020 [1]. It presents with a range of central nervous system dysfunctions, leading to diverse physical and cognitive impairments in people with MS (pwMS). These impairments include visual, oculomotor, motor, or coordination deficits, sphincter disturbances, pain, and mental or emotional changes such as fatigue, depression, and anxiety. Disease severity is commonly quantified using functional system (FS) scores, which measure impairments across eight specific domains, e.g., vision, cognition, and walking distance. The FS scores are aggregated into the Expanded Disability Status Scale (EDSS) [2], a universally applied, MS-specific measure of overall impairment and disability progression in both clinical practice and research, including treatment trials and health economic evaluations [3]. Importantly, FS scores are routinely assessed in nearly all pwMS worldwide, highlighting their potential for translating research findings into clinical practice. Their widespread use in real-world settings could ease application of insights from FS-based studies into MS care.

Health-related quality of life (HRQoL) is a multidimensional construct reflecting the physical, psychological, and social well-being of individuals and is a critical patient-reported outcome measure (PROM) in MS [4]. It provides essential insights into the broader impact of the disease and serves as a benchmark for evaluating interventions [5]. Among the available instruments for measuring HRQoL, the EuroQoL-5D-5L (EQ-5D-5L) stands out for its concise format, ease of administration, and ability to generate utility scores for health economic evaluations [6, 7]. Its generic design enables cross-disease and cross-country comparisons, making it particularly suitable for informing clinical and policy decisions in diverse healthcare settings [8, 9]. While disease-specific tools such as the Multiple Sclerosis Quality of Life-54 (MSQOL-54) [10] or the Multiple Sclerosis Impact Scale (MSIS-29) [11], may capture MS-specific challenges in more detail, their extensive formats limit their use in routine clinical practice. In contrast, the EQ-5D-5L offers a practical alternative that is well-suited for real-world application. It provides actionable insights to clinicians and healthcare systems due to its frequent use allowing comparison [12].

Although previous studies have examined the relationship between HRQoL and general disability progression in MS, such as that measured by the overall EDSS score [13, 14], this approach may oversimplify the relationship between impairment and HRQoL. Research has suggested a non-linear relationship between EDSS and HRQoL, questioning the validity of interpreting HRQoL based on aggregate EDSS scores [15]. The additive impact of specific impairments, as captured by FS scores, on HRQoL has not been thoroughly investigated [16]. While one study evaluated the additive impact of participant-rated symptom severity on HRQoL [17], others have focused on specific symptoms, such as spasticity [18] or urinary incontinence [19], without accounting for the collective effects of multiple other impairments. However, understanding the interplay of FS scores and HRQoL is critical, as FS scores are widely assessed in routine MS care and could serve as effective proxies for HRQoL when appropriately validated. Employing additive models would allow for the simultaneous evaluation of multiple impairments, enabling a more comprehensive assessment of their collective impact.

Given that assessment of FS scores and the EQ-5D-5L could be easily implemented in clinical settings, this study aimed to bridge a research gap by evaluating their relationship. Specifically, a primary analysis evaluated the association between individual FS scores and HRQoL, while a secondary analysis tested their collective association with HRQoL. By investigating the relationship of routinely assessed FS scores and a generic PROM like the EQ-5D-5fL, the results of this study could provide actionable insights for both clinical practice and policy, identifying areas for targeted intervention to improve HRQoL outcomes in pwMS.

## Methods

### Study design

This prospective cross-sectional cohort study recruited participants from the Neuroimmunology Clinic at Leipzig University Hospital between March 1st, 2023, and February 29th, 2024. Eligible participants were individuals diagnosed with MS of relapsing-remitting or progressive disease course, as per current diagnostic criteria [20]. Patients with clinically isolated syndrome or other autoimmune disorders of the central nervous system (e.g., neuromyelitis optica spectrum disorder) were not offered participation. To reduce impact of utility decrement due to relapse, participants experiencing relapse at time of or between measurements of EDSS or EQ-5D-5L were excluded [21].

Upon obtaining written informed consent, participants completed a paper-based questionnaire including the EQ-5D-5L, while EDSS assessments were conducted during routine clinical evaluations on the same day. If EDSS had not been assessed on the day of EQ-5D-5L measurement and participants had not reported any relapse or subjective change in impairment since last assessment, the most recent available EDSS from medical records was used for analysis. Additional patient data were extracted from medical records and linked with questionnaire responses and clinical assessments.

#### Ethics approval

was obtained from the institutional review board of the Medical Faculty, Leipzig University (reference number 063/23-ek), adhering to the Declaration of Helsinki guidelines. All participants provided written informed consent. The use of EQ-5D-5L was authorized by EuroQol (registration number 54658). The study was registered with the German Clinical Trial Register (reference number DRKS00031556) and followed STROBE Reporting Guidelines for cross-sectional studies [22].

### Data

HRQoL was assessed using the German version of the EQ-5D-5L questionnaire. It operationalizes information about mobility, self-care, usual activities, pain/discomfort, and anxiety/depression, with each dimension rated on a Likert scale ranging from ‘no problems’ (1) to ‘extreme problems’ (5). The respective scores were combined to generate an index value using the German standard value set, which covers an evaluation space from − 0.661 to 1 [23]. An index value of 1 encodes scores of 1 across all dimensions, i.e., 11111, indicating the best quality of life. An index value of 0 represents a health state equivalent to death, while values below 0 aim to indicate states deemed “worse than death” by societal valuation, not necessarily the mental health status of the respondents. Using the German value set, 55555 would be encoded to an index value of -0.661. Additionally, the EQ-5D-5L includes a vertical visual analogue scale (VAS) enabling patients to self-rate their overall health, ranging from ‘The best health you can imagine’ (100) to ‘The worst health you can imagine’ (0).

Assessments of impairment were conducted using the EDSS during outpatient clinic visits. The EDSS assessment includes seven FS for pyramidal, cerebellar, brainstem, sensory, bowel and bladder, visual, and cerebral (or cognitive) function, as well as walking distance as eighth component. The FS scores operationalize impairment for each functional system on an ordinal clinical rating scale ranging from 0 to 5 (cerebellar, brainstem, cerebral/cognition) or 0 to 6 [2]. While 0 represents normal function in each FS, values of 1 generally indicate an abnormal finding on neurological examination without impairment, values of 2 or 3 imply mild or moderate impairment and full scores essentially represent loss of function [24]. Walking distance was measured metrically in clinical assessment and was operationalized dichotomously (limited or unlimited) for subsequent data analyses. Following clinical convention and EDSS definition, a walking distance of more than 500 meter was coded as unrestricted [25].

Available sociodemographic and clinical features were extracted from medical records. For each variable (age, sex, disease course, disease duration, psychiatric comorbidity, somatic comorbidity, job status, relationship status), univariate linear regression was modelled with the EQ-5D-5L index value as the dependent variable and the respective factor as an independent variable. Variables with statistically significant coefficient estimates (age, disease course, psychiatric comorbidity, somatic comorbidity, job status) were used to adjust multivariate linear regression (MLR) as described below.

### Statistical analyses

Descriptive statistics, including median and interquartile range (IQR) for continuous variables, and counts and percentages for categorical data, summarized participants’ characteristics.

The primary analysis tested the hypothesis H_0_ that individual impairment domains were not unidirectionally associated with HRQoL. Eight MLR models were constructed, one for each FS and walking distance, with the respective FS as an independent ordinal predictor and the EQ-5D-5L index value as the dependent outcome variable. Discrete FS scores were coded as indicator variables, with a score of 0 serving as the reference category [26]. H_0_ was rejected if the 95% confidence intervals (95%-CI) of regression coefficients for all other levels of the indicator variable were statistically significant with the same direction of estimates.

In a second analysis, MLR was conducted with the EQ-5D-5L index value as the dependent outcome variable and FS scores as well as walking distance as standardized independent variables to assess and compare the additive impact of impairment on HRQoL. In contrast to the first analysis, FS scores were represented linearly as continuous variables. As described above, the model was also adjusted for features potentially affecting index values. Multicollinearity was assessed using the Variance Inflation Factor (VIF). 95%-CI were computed via bootstrap (10,000 random iterations with random sampling with replacement) to account for potential bias and skewness in the data, addressing violations of the homoscedasticity assumption. Results were considered statistically significant if 95%-CI did not include 0 (α = 0.05).

Statistical analyses were performed using R Statistical Software (Version 4.4.0) with R Studio (Version 2024.04.0 + 735) [27, 28].

## Results

**Fig. 1:**
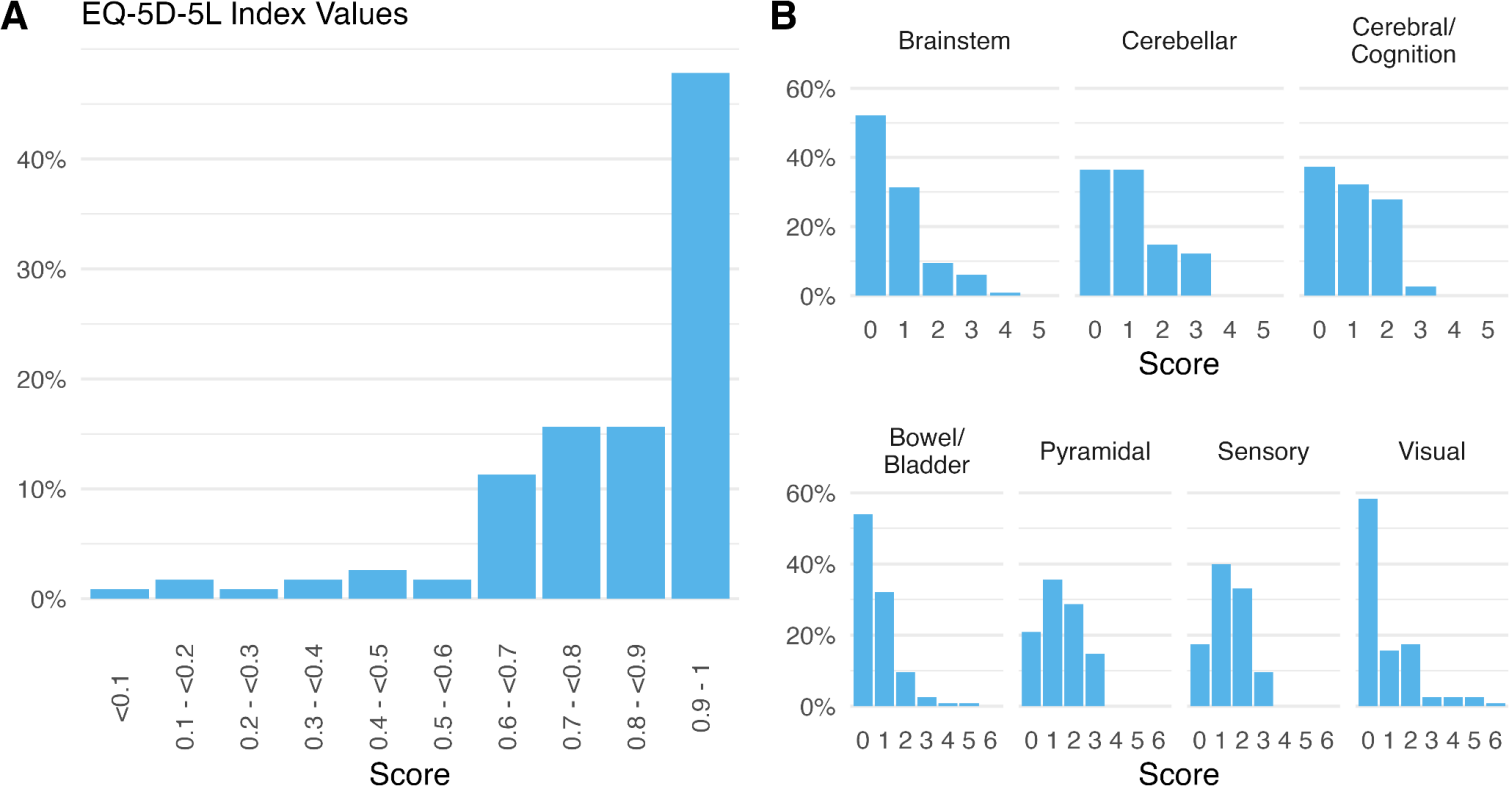
Distribution of HRQoL and disability. **A** displays the distribution of EQ-5D-5L index values assessed in patient questionnaires, with scores of 1 indicating the best quality of life and scores of 0 representing health states equivalent to death. **B** shows the distribution of FS scores ranging from 0 for normal function to maximum possible scores of 5 (top row) or 6 (bottom row) representing full loss of function. Abbreviations: FS = functional system; EDSS = Expanded disability status scale

Of 196 pwMS invited to participate in this study, 119 (60.71%) responded to the questionnaires. After imputing one missing EQ-5D-5L item by copying the respective entry of the most similar subject, complete data from 115 participants were included for analyses. In median (IQR) 0 days (23 days) had passed between assessment of EDSS and EQ-5D-5L. Table 1 summarizes the clinical and sociodemographic characteristics of the cohort. Overall, 35 (30.43%) participants had an EDSS of ≤ 1.5, 61 (53.04%) had scores between 2 and 4, and 19 (16.52%) had scores ≥ 4.5. Limited walking distance was observed in 56 (48.70%) participants. Distribution of FS scores is shown in Fig. 1B and C.

**Table 1:** Study cohort characteristics (*N* = 115)

Characteristics		Value	
**Age**, years		40.90	(33.65 – 50.60)
**Sex**, female		74	(64.35%)
**Disease course**			
	RRMS	96	(83.48%)
	SPMS	4	(3.48%)
	PPMS	15	(13.04%)
**Disease duration**, years		6.94	(4.02 – 13.80)
**DMT**			
	Highly effective	63	(54.78%)
	Moderately effective	20	(17.39%)
	Modestly effective	11	(9.57%)
	Others	7	(6.09%)
	None	14	(13.04%)
**Comorbidity**			
	Somatic	86	(74.78%)
	Psychiatric	30	(26.09%)
	None	25	(21.74%)
**Working status**			
	Full time employment	44	(38.26%)
	Part time employment	29	(25.22%)
	Disability retirement	24	(20.87%)
	Unemployed	6	(5.22%)
	Others	12	(10.43%)
**Relationship status**			
	In relationship	74	(64.45%)
	Single	37	(32.17%)

Data are median (interquartile range) or n (% of entire study population). Abbreviations: RRMS = relapsing remitting multiple sclerosis; SPMS = secondary progressive multiple sclerosis; PPMS = primary progressive multiple sclerosis; DMT = disease modifying therapy category according to current international guidelines (e.g., Hauser SL, Cree BAC. Treatment of Multiple Sclerosis: A Review. Am J Med. 2020)

The EQ-5D-5L index value, representing overall HRQoL, ranged from − 0.12 to 1, with a median of 0.87 and an IQR of 0.76 to 0.97 (Fig. 1A). Participants’ self-rated overall health, measured using the EQ-5D-5L VAS, had a median score of 75 (IQR 60.00–87.75), with 3 (2.61%) missing responses.

Primary analysis examined the association between individual FS and EQ-5D-5L index values. Increased scores in brainstem, pyramidal, cerebellar, sensory, and cerebral/cognitive FS were associated with decreasing EQ-5D-5L index values (Table 2). Additionally, limited walking distance was associated with a reduction of -0.22 (95%-CI -0.29 – -0.16) in the EQ-5D-5L index value. Not all coefficient estimates for categorical variables of visual and bladder/bowel function were statistically significant.

**Table 2:** Primary analyses – association of HRQoL and each EDSS functional system score

FS	visual				brainstem				pyramidal			
Score	Estimate	95%-CI			Estimate	95%-CI			Estimate	95%-CI		
1	-0.040	-0.129	–	0.037	**-0.147**	**-0.245**	**–**	**-0.077**	**-0.049**	**-0.098**	**–**	**-0.005**
2	**-0.157**	**-0.326**	**–**	**-0.046**	**-0.245**	**-0.420**	**–**	**-0.112**	**-0.233**	**-0.341**	**–**	**-0.171**
3	0.034	-0.076	–	0.114	**-0.206**	**-0.316**	**–**	**-0.113**	**-0.315**	**-0.431**	**–**	**-0.226**
4	**-0.188**	**-0.457**	**–**	**-0.035**	**-0.200**	**-0.230**	**–**	**-0.151**	n.a.			
5	**0.096**	**0.038**	**–**	**0.166**	n.a.				n.a.			
6	-0.002	-0.037	–	0.045	n.a.				n.a.			
**FS**	**cerebellar**				**sensory**				**bowel/bladder**			
Score	Estimate	95%-CI			Estimate	95%-CI			Estimate	95%-CI		
1	**-0.062**	**-0.110**	**–**	**-0.015**	**-0.078**	**-0.136**	**–**	**-0.014**	**-0.143**	**-0.225**	**–**	**-0.079**
2	**-0.318**	**-0.445**	**–**	**-0.210**	**-0.152**	**-0.255**	**–**	**-0.070**	**-0.340**	**-0.571**	**–**	**-0.195**
3	**-0.301**	**-0.498**	**–**	**-0.201**	**-0.290**	**-0.446**	**–**	**-0.167**	-0.060	-0.115	–	0.031
4	n.a.				n.a.				**-0.278**	**-0.306**	**–**	**-0.241**
5	n.a.				n.a.				0.057	-0.029	–	0.094
**FS**	**cerebral/cognition**								**Walking distance**			
Score	Estimate	95%-CI							(reference “unlimited”)			
1	**-0.170**	**-0.249**	**–**	**-0.115**		Score			Estimate	95%-CI		
2	**-0.257**	**-0.324**	**–**	**-0.199**		“limited”			**-0.216**	**-0.287**	**–**	**-0.159**
3	**-0.463**	**-1.087**	**–**	**-0.140**								
4	n.a.											
5	n.a.											

Coefficient estimates for change in EQ-5D-5L index value derived from multivariate linear regression with FS scores as categorial indpendent variable. Estimates for each change in FS score are referencing to FS of 0. Statistically significant estimates are bold. FS with significant estimates for each score suggest significant overall association between the respective FS and index values. Abbreviations: FS = functional system; 95%-CI = bootstrapped 95% confidence interval; n.a. = no data available in study cohort or score not existent in functional system

**Fig. 2:**
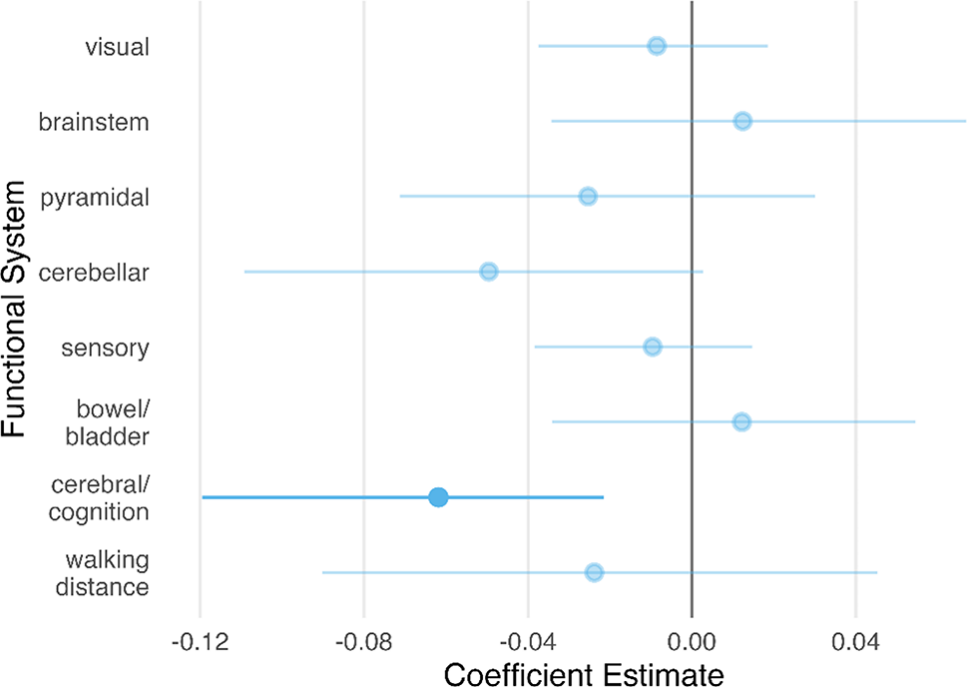
Association of functional systems and HRQoL. Normalized coefficient estimates and 95%-CI of each FS with EQ-5D-5L index value as the dependent variable. Abbreviations: FS = functional system; 95%-CI = 95% confidence interval

Subsequent analysis assessed the additive impact of physical and cognitive impairments on HRQoL using MLR (Fig. 2). After bootstrapping the adjusted model, decreased cerebral/cognitive function remained significantly associated with reduced EQ-5D-5L index values (β = -0.062, 95%-CI -0.122 – -0.023), showing numerically strongest impact among all FS. Moreover, 95%-CI of this FS included values of minimal clinically important difference reported for a German population [29]. A VIF of 3.095 for the cerebellar FS and VIFs below 3 for all other predictors indicated that no substantial collinearity was present among the variables. The adjusted model explained approximately 46% of the variability in EQ-5D-5L index values (R² = 0.457, F(13,101) = 9.387, *p* < 0.001).

## Discussion

This cross-sectional cohort study investigated the connection between individual MS-related impairments and HRQoL by analyzing the association of EDSS FS scores with EQ-5D-5L index values. Unadjusted analyses revealed significant associations between brainstem, pyramidal, cerebellar, sensory, and cerebral/cognitive dysfunctions and reduced HRQoL in pwMS. Among these, cerebral/cognitive dysfunction exhibited the strongest effect. In adjusted analyses accounting for covariates, including psychiatric comorbidities, only cerebral/cognitive dysfunction retained a significant association, with a one standard deviation decrease in cognitive function corresponding to a 6% reduction in HRQoL.

The inclusion of a representative cohort of pwMS with 83% of participants exhibiting a relapsing-remitting disease subtype aligns with real-world distributions [30, 31], while a questionnaire return rate of 60% suggests high practicality of the EQ-5D-5L in routine clinical settings. The HRQoL distribution in this cohort resembles that of the German general elderly population (aged 65 years and older), with slight differences likely attributable to the younger age of pwMS in this study [32].

Contrary to survey results among pwMS [33], the association of bladder/bowel FS with HRQoL was not statistically significant, potentially reflecting limited interference with activities of daily life in this cohort. Similarly, the absence of a significant association with visual FS likely stems from the high proportion of participants with normal visual function, represented by a strong skew in this FS distribution.

Consistent with prior research, our findings reaffirm the negative association of cognitive impairments with HRQoL in pwMS [13, 14, 34]. However, the observed effect in this cohort may partially reflect the formal definition of cerebral function within the EDSS. Originally established by John F. Kurtzke in 1965, cerebral function scoring ranged from “mood alteration only” (score 1) to “dementia or chronic brain syndrome” (score 5), with intermediate scores reflecting progressive “decrease in mentation” [2, 35]. This definition, unchanged since its integration into the EDSS in 1983 [2], permits variability in interpretation and lacks specific guidance on assessing cognitive function in the presence of psychiatric comorbidities. For example, personality disorders and substance abuse, observed in our cohort, may not be adequately captured by the current FS. Updating the EDSS to better account for the full spectrum of cognitive and psychiatric impairments could improve its utility in clinical practice.

Despite this constraint, our study adds valuable insights by using the EDSS FS – a widely established, clinician-reported measure – to explore the relationship between impairment and HRQoL. Unlike prior studies relying on only partially validated patient-reported measures not commonly used in non-research settings, such as the MS Symptom Score [17, 36], our findings facilitate translation into clinical practice and underscore the relevance of cognitive dysfunction in pwMS. Our results also support the hypothesis that cognitive impairments in pwMS substantially affect daily living, contributing to high unemployment rates, reduced income, and poorer performance in activities such as driving, financial management, and household tasks [37]. This emphasizes the need for regular assessments to detect cognitive deficits and target interventions to address patients’ needs.

Current MS assessment and management strategies have predominantly focused on physical impairment and relapse reduction, with less emphasis on cognitive dysfunction [3, 38]. However, recognizing cognitive impairment as a significant determinant of HRQoL could shift priorities toward detecting cognitive deficits and preserving cognitive function. Although the respective FS of the EDSS offers only an approximate measure of cognition, it could serve as a useful indicator to encourage clinicians to refer patients for more comprehensive cognitive evaluations. Established tools for the assessment of cognition such as the Brief International Cognitive Assessment for MS (BICAMS) or the Minimal Assessment of Cognitive Function in MS (MACFIMS) are well-suited to facilitate early detection and intervention for cognitive deficits [39, 40, 41]. While both tests have been validated for their reliability and sensitivity in detecting cognitive impairment in pwMS, the Montreal Cognitive Assessment (MoCA) is often favored for its brevity and ease of administration, emphasizing the need for quick and reliable screening methods in routine evaluations [42, 43, 44]. Although the MUSIC [45] has been suggested as a screening tool in pwMS, its use has been mostly limited to Germany, and the Symbol Digit Modalities Test (SDMT) remains the most frequently used tool for monitoring cognitive decline in pwMS [46].

When exhibiting cognitive deficits, promising approaches to address these include cognitive rehabilitation programs, such as computer-based cognitive training and memory enhancement therapies, which target specific cognitive domains [39, 47]. Additionally, recent pharmacological studies have suggested that disease-modifying therapies may have positive effects on cognitive function in pwMS [48].

Through a robust statistical methodology and thorough discussion of the results seen in this representative cohort, we provide solid information on the effects of impairment on HRQoL in pwMS. Employing an established, clinician-rated measure for impairment, we add valuable information with possible consequences for clinical routine. Nevertheless, several limitations must be considered. The relatively small, single-center cohort with moderate disability may limit generalizability. The low number of pwMS with high FS scores could have impacted analyses. Although collinearity was not substantial, as reflected by VIF values below conventional thresholds, some degree of multicollinearity may have reduced precision. Cognitive dysfunction was assessed via EDSS, yet additional cognitive measures could enhance monitoring [49]. We did not record time since last relapse or assess age-related frailty, both of which may influence HRQoL and EDSS scores independent of MS-related disability [21, 50]. This adds to research emphasizing the influence of factors other than MS-induced impairment on MS-specific disability scores such as EDSS [51]. Despite its practicality, the EQ-5D-5L might have failed to capture the entire range of attributes of HRQoL [52]. Alternative utility measures such as the EQ-5D-5L-Psychosocial, which incorporates additional psychosocial questions, could have performed better in our cohort of pwMS with no to moderate disability [53]. Converting ordinal FS scores into continuous variables may have introduced measurement error, and obtaining EQ-5D-5L and EDSS data at different time points could have introduced bias. Thus, despite having demonstrated relevant and translatable associations, the study’s results demand reproduction in a larger cohort.

In conclusion, this study underscores the critical role of cognitive dysfunction in determining HRQoL in pwMS. Despite the limitations of the EDSS, its FS scores provide a useful, universally available indicator to identify patients who may benefit from comprehensive cognitive evaluations. A pro-active integration of cognitive assessments and targeted interventions into clinical routine could have the potential to improve outcomes and overall well-being for pwMS. These findings contribute to a growing body of evidence emphasizing the importance of cognitive health in pwMS and highlight the need for effective screening and therapeutic strategies.

## Electronic supplementary material

Below is the link to the electronic supplementary material.

Supplementary Material 1
